# Longitudinal cognitive decline characterizes the profile of non-PD-manifest *GBA1* mutation carriers

**DOI:** 10.1038/s41531-024-00706-1

**Published:** 2024-04-22

**Authors:** Benjamin Roeben, Inga Liepelt-Scarfone, Stefanie Lerche, Milan Zimmermann, Isabel Wurster, Ulrike Sünkel, Claudia Schulte, Christian Deuschle, Gerhard W. Eschweiler, Walter Maetzler, Thomas Gasser, Daniela Berg, Kathrin Brockmann

**Affiliations:** 1 Center of Neurology, Department of Neurodegeneration and Hertie-Institute for Clinical Brain Research, University of Tübingen, Hoppe-Seyler-Str. 3, 72076 Tübingen, Germany; 2 German Center for Neurodegenerative Diseases, University of Tübingen, Otfried-Müller-Str. 27, 72076 Tübingen, Germany; 3 IB-Hochschule, Stuttgart, Germany; 4 Department of Psychiatry and Psychotherapy, University of Tübingen, Calwer Str. 14, 72076 Tübingen, Germany; 5 Geriatric Center at the University Hospital of Tübingen, Calwer Str. 14, 72076 Tübingen, Germany; 6 Department of Neurology, University Hospital Schleswig-Holstein, Campus Kiel, and Kiel University, Arnold-Heller-Straße 3, 24105 Kiel, Germany

**Keywords:** Parkinson's disease, Diagnostic markers

## Abstract

With disease-modifying treatment for Parkinson’s disease (PD) associated with variants in the glucocerebrosidase gene (*GBA1*) under way, the challenge to design clinical trials with non-PD-manifest *GBA* mutation carriers (GBA1_NMC_) comes within close reach. To delineate trajectories of motor and non-motor markers as well as serum neurofilament light (sNfL) levels and to evaluate clinical endpoints as outcomes for clinical trials in GBA1_NMC_, longitudinal data of 56 GBA1_NMC_ carriers and 112 age- and sex-matched *GBA1* wildtype participants (GBA1_wildtype_) with up to 9 years of follow-up was analyzed using linear mixed-effects models (LMEM) and Kaplan–Meier survival analysis of clinical endpoints for motor and cognitive function. GBA1_NMC_ showed worse performance in Pegboard, 20 m fast walking, global cognition as well as in executive and memory function at baseline. Longitudinally, LMEM revealed a higher annual increase of the MDS-UPDRS III bradykinesia subscore in GBA1_NMC_ compared to GBA1_wildtype_, but comparable trajectories of all other motor and non-motor markers as well as sNfL. Kaplan–Meier survival analysis showed a significantly earlier progression to clinical endpoints of cognitive decline in GBA1_NMC_. Incidence of PD was significantly higher in GBA1_NMC_. In conclusion, our study extends data on GBA1_NMC_ indicating early cognitive decline as a potentially characteristic feature. Comprehensive longitudinal assessments of cognitive function are crucial to delineate the evolution of early changes in GBA1_NMC_ enabling a more accurate stratification and allow for a more precise definition of trial design and sample size.

## Introduction

It is well established that the classical motor manifestation of Parkinson’s disease (PD) is preceded by a phase which is characterized by the occurrence of several non-motor and early motor signs^1^. Non-motor symptoms include amongst others REM sleep behavioral disorder (RBD), hyposmia, autonomic dysfunction and neuropsychiatric symptoms such as depression and cognitive dysfunction whereas reduced arm swing and bradykinesia indicate early motor signs. However, kind and prevalence of these symptoms as well as time of occurrence and progression in relation to the onset of the classical motor manifestation is highly variable among individuals. With disease-modifying treatment options targeting different disease-specific pathways at hand, this poses challenges for designing clinical trials: *Who* should enter such clinical trials, *when* is the best time-point, *how long* should the intervention take, and *what* might be reasonable outcome measures^2^. Individuals with genetic mutations represent a valuable subgroup with a defined risk and known underlying pathophysiology for the development of PD. However, mutations in genes with high penetrance such as *SNCA* or bi-allelic *PRKN* and *PINK1* are rare and thereby limiting the sample size whereas genes with more common mutations such as *LRRK2* and *GBA1* show reduced and age-dependent penetrance. Therefore, detailed longitudinal evaluation of clinical trajectories is needed in order to determine effect sizes of different assessments and biomarkers.

Heterozygous mutations in the *GBA1* gene represent the most important genetic risk factor for Parkinson disease (PD) and dementia with Lewy Bodies (DLB)^3^ with reasonable prevalence, penetrance and occurrence across different populations. Clinically, people with PD carrying heterozygous *GBA1* mutations (GBA1-PD) show more severe trajectories with faster progression of motor and non-motor impairment^4,5^, specifically more rapid and earlier development of cognitive decline^6–8^ compared to PD without *GBA1* mutation. Importantly, this clinical phenotype is dependent on the *GBA1* genotype with severe mutations predisposing to more prominent motor impairment and cognitive decline as compared to *GBA1* risk variants and mild mutations^4,6,9,10^.

Given the prominent findings from the manifest disease phase, smaller studies have focused on non-PD-manifest *GBA1* mutation carriers who did not meet diagnostic criteria for manifest PD or DLB at time of assessment (GBA1_NMC_)^11^. Longitudinal analysis over 2 and 6 years found greater deterioration in scales of depression, RBD, olfaction, global cognition as well as the Unified Parkinson’s Disease Rating Scale (UPDRS) part II and III scores in the GBA1_NMC_ group compared to healthy controls without *GBA1* mutation (GBA1_wildtype_)^12–14^. Focusing on a more detailed investigation of cognition, a cross-sectional study has recently shown that executive function assessed by the Stroop test was worse in GBA1_NMC_ compared to GBA1_wildtype_ and that reduced global cognitive function assessed with the Montréal Cognitive Assessment (MoCA) clustered with hyposmia. Verbal memory, overall motor score, presence of RBD or depression were similar between groups^15^. However, with clinical trials using disease-modifying compounds for PD at the horizon, there is still an urgent need for more sensitive and quantitative progression markers. Addressing this issue, we investigated trajectories of quantitative motor and non-motor parameters leveraged by a comprehensive assessment battery as well as serum levels of neurofilament light chain (sNfL) in *GBA1* carriers compared to age- and sex-matched healthy controls in a prospective longitudinal study with up to 9 years of follow-up.

## Results

### Baseline characteristics

Details on demographic characteristics and frequencies of the different *GBA1* variants are shown in Table 1. The total cohort (*n* = 168) included 762 assessments (*n* = 164 with 1–4 follow-up assessments) with a mean follow-up time of 6.3 ± 2.0 years in the GBA1_NMC_ group versus 7.7 ± 1.2 years in the GBA1_wildtype_ group. The 56 *GBA1*_NMC_ accounted for a prevalence of 4.7% in the overall TREND study. GBA1_wildtype_ and GBA1_NMC_ were similar in sex (female 50.9% and 51.8%; *p* = 0.913) and mean age (63 years both groups, *p* = 0.982). There was a trend of a more frequent family history for PD in the GBA1_NMC_ group (25.0% vs 13.4% *p* = 0.061), while a family history for dementia was more frequent in the GBA1_wildtype_ group (55.4% vs 33.4%; *p* = 0.002). Years of education were higher in the GBA1_wildtype_ group (mean years of education: 14.5 ± 2.3 years vs 13.5 ± 3.1 years; *p* = 0.041).

**Table 1:** Demographic characteristics, prodromal, motor, non-motor and fluid biomarkers of GBA1 mutation carriers compared to age- and sex-matched GBA1_wildtype_ and PD phenoconverters

	GBA1 _wildtype_ (*n* = 112)	GBA1 _mutation_ (*n* = 56)	*p*	*PD converter*	
GBA1 variant, *n* (PDc)		GBA1_risk_ E365K, 19 (2 PDc) T408M, 26 (1 PDc) T336S, 1 N427K, 1 N427K + T408M, 1 GBA1_mild_ N409S, 5 (2 PDc) GBA1_severe_ H294Q, 1 L483P, 1 L483P + E365K, 1		GBA1_wildtype_ (*n* = 2)	GBA1_mutation_ (*n* = 5) GBA1risk E365K, 2 T408M, 1 GBA1_mild_ N409S, 2
*Demographics*				*Assessment at baseline*	
Females, *n* (%)	57 (50.9)	29 (51.8)	0.913	1 (100)	1 (25.0)
Age, years	63.3 (7.4)	63.4 (7.5)	0.982	73.0	66.8 (5.9)
Handedness, right/left/ambidextrous	100/1/11 (89.3/0.9/9.8)	47/2 /7 (83.9/3.6/12.5)	0.459	2/0/0 (100.0/0/0)	4/0/1 (80.0/0/20.0)
Family history of PD, *n* (%)	15 (13.4)	14 (25.0)	***0.061***	0(0)	2 (40.0)
Family history of dementia, *n* (%)	62 (55.4)	17 (30.4)	**0.002**	1 (100)	1 (20.0)
Years of education, years	14.5 (2.3)	13.5 (3.1)	**0.041**	15.0	14.8 (4.5)
Drop-outs per follow up, *n* (%)					
*Follow-up 1 (year 2)*	3 (2.7%)	1 (1.8%)	0.720	0 (0%)	1 (25.0%)
*Follow-up 2 (year 4)*	4 (3.6%)	1 (1.8%)	0.521	0 (0%)	0 (0%)
*Follow-up 3 (year 6)*	9 (8.0%)	0 (0%)	**0.029**	0 (0%)	2 (50.0%)
*Follow-up 4 (year 8)*	5 (4.5%)	7 (12.5%)	***0.057***	0 (0%)	0 (0%)
*Prodromal markers*					
BDI II	8.9 (8.0; MV 2)	7.7 (7.6; MV 1)	0.353	2.0	2.0 (2.7)
RBDSQ	2.7 (2.3)	2.5 (2.2)	0.485	3.0	3.3 (4.0)
Olfaction (16 Sniffin’ Sticks)	10.9 (2.8)	11.5 (3.2)	0.211	4.0	7.3 (2.9)
Orthostatic dysfunction (UMSARS item 9)	0.3 (0.5; MV 1)	0.2 (0.4)	0.145	0(0)	1 (25.0)
Urinary dysfunction (UMSARS item 10)	0.5 (0.6; MV2)	0.5 (0.7)	1.000	1 (100)	1 (25.0)
Sexual dysfunction (UMSARS item 11)	0.9 (1.3; MV 4)	1.1 (1.4)	0.301	1 (100)	2 (50.0)
Bowel dysfunction (UMSARS item 12)	0.2 (0.5; MV 2)	0.1 (0.3)	0.177	0(0)	1 (25.0)
*Motor function*					
MDS-UPDRS III total score	1.9 (2.5)	1.7 (2.1)	0.564	5.0	3.8 (4.4)
*Tremor subscore*	0.5 (1.5)	0.3 (1.0)	0.240	3.0	1.5 (3.0)
*Rigidity subscore*	0.1 (0.4)	0.1 (0.4)	0.504	2.0	0(0)
*Bradykinesia subscore*	1.0 (1.5)	1.1 (1.6)	0.451	0	2.0 (1.8)
*PIGD subscore*	0.1 (0.4)	0.1 (0.4)	0.890	0	0.3 (0.5)
Pegboard right hand, seconds	13.8 (1.7; MV 1)	14.4 (1.9; MV 4)	**0.033**	13.3	14.5 (2.4)
Pegboard left hand, seconds	13.4 (1.6)	13.9 (2.0; MV 4)	0.103	13.3	14.3 (2.6)
Pegboard simultaneous, seconds	11.1 (1.5; MV 5)	11.5 (1.8; MV 4)	0.117	10.0	12.0 (2.0)
3 m Timed-Up-&-Go right foot first, seconds	10.8 (1.9; MV 1)	9.9 (1.9; MV 2)	**0.008**	10.2 (0.1)	9.9 (0.3; MV: 1)
3 m Timed-Up-&-Go left foot first, seconds	10.0 (1.5; MV 1)	9.5 (1.7; MV 2)	0.113	10.9 (0.5)	9.6 (0.5; MV: 1)
20 m normal walking right foot first, seconds	14.6 (2.1; MV1)	15.2 (2.1; MV 2)	0.092	14.5 (1.1)	15.3 (1.3; MV: 1)
20 m normal walking left foot first, seconds	14.9 (1.9; MV 1)	15.4 (2.2; MV 2)	0.094	15.0 (1.1)	15.4 (1.5; MV: 1)
20 m fast walking right foot first, seconds	11.8 (1.9; MV 1)	12.6 (2.6; MV 2)	**0.025**	10.8 (2.3)	12.5 (1.2; MV: 1)
20 m fast walking left foot first, seconds	11.9 (2.1; MV 2)	12.6 (2.4; MV 2)	***0.067***	10.8 (1.5)	12.5 (1.3; MV: 1)
20 m fast walking & crosses, seconds	13.5 (2.0; MV 1)	14.2 (2.6; MV 2)	***0.059***	13.6 (1.1)	13.7 (1.1; MV: 1)
20 m fast walking & subtractions, seconds	14.6 (3.0; MV 1)	15.4 (3.1; MV 2)	0.122	13.8 (0.8)	14.9 (1.6; MV: 1)
*Cognition*					
MMSE score	28.9 (1.1)	28.3 (1.2)	**0.001**	28.5 (0.7)	28.0 (2.0)
MoCA score	25.6 (2.6)	24.1 (2.7)	**0.001**	24.5 (2.1)	23.8 (4.2)
CERAD-Plus sum score	85.3 (6.4)	82.9 (7.8)	**0.036**	81.5 (3.5)	82.2 (8.7)
*Word list learning*	21.5 (3.3)	20.5 (3.3)	***0.065***	17.0 (2.8)	21.2 (2.6)
*Word list recall*	7.5 (1.7)	7.3 (1.8)	0.366	5.5 (2.1)	7.2 (1.9)
*Word list recognition correct*	9.8 (0.5)	9.6 (0.7)	0.172	10.0(0)	9.6 (0.6)
*Word list recognition incorrect*	10.0 (0.2)	10.0 (0.2)	0.750	10.0(0)	10.0(0)
*Word list discriminability*	98.7 (2.8)	97.9 (4.0)	0.183	100.0(0)	98.0 (2.7)
*Figure recall*	9.3 (2.0)	8.5 (2.2)	**0.031**	9.5 (0.7)	7.2 (3.4)
*Figure drawing*	10.5 (0.8)	9.9 (1.4)	**0.004**	11.0(0)	9.2 (2.2)
*Semantic verbal fluency*	23.9 (6.0)	23.2 (5.7)	0.463	24.5 (3.5)	24.4 (7.8)
*Phonematic verbal fluency*	18.1 (5.2 MV 35)	15.4 (6.1; MV 7)	**0.007**	17.0 (5.7)	17.5 (5.8)
*Boston naming Test*	14.6 (0.7)	14.4 (1.0)	0.104	15.0(0)	14.0 (1.2)
*TMT-A*	36.7 (12.1; MV 1)	40.2 (12.2)	***0.073***	62.5 (3.5)	33.6 (8.2)
*TMT-B*	88.4 (33.1)	93.5 (43.9; MV 2)	0.407	85.5 (34.7)	87.6 (40.2)
*TMT B-A*	52.0 (29.2; MV 1)	54.6 (38.7; MV 2)	0.636	23.0 (38.2)	54.0 (34.3)
*TMT B:A*	2.5 (0.9; MV 1)	2.4 (0.8; MV 2)	0.420	1.4 (0.6)	2.6 (0.8)
*Fluid Biomarkers*					
Serum Neurofilament light, pg/ml	15.2 (11.6; MV 2)	13.8 (5.3; MV 1)	0.373	14.1 (1.9)	15.8 (4.3; MV: 1)

Demographic characteristics, prodromal, motor and non-motor markers, and serum neurofilament light levels of *GBA1* mutation carriers compared to age- and sex-matched GBA1_wildtype_ (2:1-Matching) and PD phenoconverters. Naming of *GBA1* variants is based on the new nomenclature for GBA variants including the 39-aminoacid residue. Values are depicted as mean with standard deviation in brackets. Student’s *t* test was used for continuous data and *χ* ^2^ test was used for categorial data. Two-sided *p* < 0.05 are presented in bold, trends with two-sided *p* < 0.1 are presented in italicized bold font.

*PDc* PD converter, *MV* Missing Values.

There were no significant differences with regard to severity of known non-motor symptoms (BDI II, RBDSQ, Sniffin Sticks, UMSARS: orthostatic, urinary, sexual, bowel dysfunction) between the *GBA1*_wildtype_ and the *GBA1*_*NMC*_ group (for details see Table 1).

In terms of motor function, *GBA1*_NMC_ performed worse in the Purdue Pegboard test with the right hand (*p* = 0.033) and in fast walking of a 20 m distance starting with the right foot (*p* = 0.025) than the GBA1_wildtype_ group, while there were trends in a similar direction for fast walking of the 20 m distance starting with the left foot (*p* = 0.067) and for fast walking while drawing crosses (*p* = 0.059) (Table 1). No differences were seen in mean MDS-UPDRS III total and sub-items scores (for details see Table 1).

*GBA1*_*NMC*_ showed worse mean MMSE and MoCA scores compared to the GBA1_wildtype_ group (both *p* < 0.001). The GBA1_NMC_ group also showed significantly lower scores in the CERAD-Plus sum score (*p* = 0.036) as well as in the CERAD-Plus subtest scores for figure drawing (*p* = 0.004), figure recall (*p* = 0.031), and phonematic verbal fluency (*p* = 0.007). Similarly, they tended to perform worse in word list learning (*p* = 0.065) and in the TMT-A (*p* = 0.073).

Mean sNfL levels did not show significant differences between the GBA1_wildtype_ and the GBA1_NMC_ group at baseline (*p* = 0.373).

### Longitudinal analyses

LMEM showed a significantly higher annual increase of the MDS-UPDRS III bradykinesia subscore of the GBA1_NMC_ group compared to the GBA_wildtype_ group (+1.13, 95% CI: −0.01–+2.26, *p* = 0.048; Table 2), but just missed significance level after adding age as a fixed factor remaining as a trend (+1.11, 95% CI: −0.06–+2.22, *p* = 0.051). All other non-motor and motor markers as well as serum NfL levels did not show significantly different slopes of GBA1_NMC_ compared to GBA_wildtype_ (for details see Table 2). However, adding age as a fixed factor in the model also revealed significant effects of age on several markers (Table 2).

**Table 2:** Linear mixed effect models of trajectories of prodromal, motor, non-motor and fluid biomarkers comparing asymptomatic GBA1 wildtype and GBA1 mutation carriers

Trajectory trend time × group interaction		Age effects
GBA1_wildtype_ vs GBA1_mutation_		p
*Prodromal markers*		
BDI II	B = −2.19 (−4.68, +0.30) *p* 0.084	0.789
RBDSQ	B = +0.26 (−0.55, +1.06) *p* 0.528	0.423
Olfaction (16 Sniffin’ sticks)	B = +0.18 (−0.91, +1.26) *p* 0.748	**<0.001**
Orthostatic dysfunction (UMSARS item 9)	B = +0.14 (−0.06, +0.34) *p* 0.157	0.813
Urinary dysfunction (UMSARS item 10)	B = −0.04 (−0.27, +0.19) *p* 0.718	**0.003**
Sexual dysfunction (UMSARS item 11)	B = −0.06 (−0.18, +0.07) *p* 0.379	**0.012**
Bowel dysfunction (UMSARS item 12)	B = −0.04 (−0.27, +0.19) *p* 0.718	**0.011**
*Motor function*		
MDS-UPDRS III total score	B = +1.09 (−0.75, +2.49) *p* 0.244	**<0.001**
*Tremor subscore*	B = −0.29 (−1.14, +0.56) *p* 0.499	**0.008**
*Rigidity subscore*	B = −0.01 (−0.21, +0.19) *p* 0.927	0.223
*Bradykinesia subscore*	**B** = **+1.13 (−0.01**, + **2.26)** ***p*** **0.048**	**<0.001**
*PIGD subscore*	B = +0.13 (−4969.88, +4969.61) *p* 1.000	0.465
Pegboard right hand, seconds	B = +1.41 (−0.90, +3.71) *p* 0.228	**<0.001**
Pegboard left hand, seconds	B = +0.76 (−1.19, +2.70) *p* 0.442	**<0.001**
Pegboard simultaneous, seconds	B = +2.41 ( + 1.00, +3.81) *p* 1.000	**<0.001**
3 m Timed-Up-&-Go, seconds	B = +0.77 (−2.13, +3.67) *p* 0.600	**<0.001**
Normal walking speed 20 m, seconds	B = −0.56 (−1.63, +0.50) *p* 0.298	**<0.001**
Fast walking speed 20 m, seconds	B = +34.13 (−3866.30, +3934.56) *p* 0.986	**<0.001**
Fast walking speed 20 m + crosses, seconds	B = +0.33 (−0.96, +1.62) *p* 0.616	1.000
Fast walking speed 20 m + subtractions, seconds	B = +1.28 (−0.38, +2.94) *p* 0.130	0.939
*Cognition*		
MMSE total score	B = +0.52 (−3.51, +4.55) *p* 1.000	1.000
MoCA total score	B = +1.08 (−0.43, +2.60) *p* 0.160	**<0.001**
CERAD-Plus sum score	B = +0.51 (−2.65, +3.66) *p* 0.752	**<0.001**
CERAD-Plus subtests		
*Word list learning sum*	B = +0.08 (−1.79, +1.62) *p* 0.923	**<0.001**
*Word list recall*	B = +0.08 (−0.79, +0.96) *p* 0.857	**<0.001**
*Word list recognition correct*	B = −0.75 (−2.59*E8, +2.59*E8) *p* 1.000	**<0.001**
*Word list recognition incorrect*	B = +0.03 (−0.22, +0.28) *p* 0.794	0.148
*Word list discriminability*	B = −0.61 (−2.55, +1.34) *p* 0.539	**0.006**
*Figure recall*	B = −0.66 (−1.54, +0.22) *p* 0.143	**<0.001**
*Figure drawing*	B = −0.07 (−0.63, +0.49) *p* 0.799	0.035
*Semantic verbal fluency*	B = +0.95 (−1.72, +3.62) *p* 0.483	0.003
*Phonematic verbal fluency*	B = +1.61 (−0.63, +3.86) *p* 0.158	0.987
*Boston Naming Test*	B = +0.14 (−0.16, +0.45) *p* 0.350	**<0.001**
*TMT A*	B = +0.42 (−6.14, +7.00) *p* 0.899	**<0.001**
*TMT B*	B = −0.68 (−17.39, +16.03) *p* 0.936	1.000
*TMT B-A*	B = −0.78 (−17.48, +15.92) *p* 0.927	**<0.001**
*TMT B/A*	B = −0.11 (−0.59, +0.37) *p* 0.645	0.017
*Fluid Biomarkers*		
Serum Neurofilament light	B = −3.35 (−12.37, +5.67) *p* 0.464	**<0.001**

The GBA1_wildtype_ group represents the reference condition. Mixed effects models were adjusted for age and years of education as appropriate. Effects of age are presented in a separate column after including age as a fixed factor in the model. All statistically significant differences (*p* < 0.05) are presented in bold. B = coefficient.

Although a formal statistical analysis of groups stratified by *GBA1* mutation severity was not possible due to small group sizes, exploratory descriptive analysis of trajectories showed steeper slopes of the MDS-UPDRS III total score as well as subscores for tremor, rigidity and bradykinesia in the GBA1_mild_ and to a lesser extent in the GBA1_risk_ group, while slopes of the MMSE, MoCA, CERAD-Plus and sNfL levels rather developed in parallel comparing GBA1_risk_, GBA1_mild_ and GBA1_severe_ with their respective age- and sex-matched GBA1_wildtype_ groups.

**Fig. 1:**
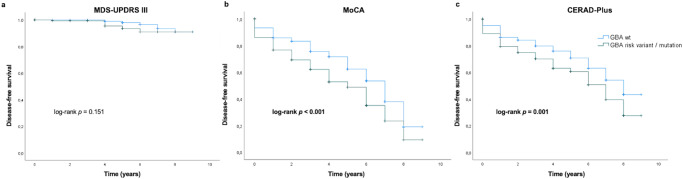
Kaplan–Meier survival analysis for clinical endpoints of motor and cognitive function. Kaplan–Meier survival analysis with log rank test and Cox Regression analysis adjusted for age show that the asymptomatic GBA_mutation_ group reach clinical endpoints for cognitive decline earlier than the GBA_wildtype_ group (clinical endpoint of motor function based on the MDS-UPDRS III (**a**); clinical endpoints of cognitive function based on cut-offs for MCI established for the MoCA total score (**b**) and the CERAD-Plus battery (**c**)).

Kaplan–Meier survival analysis with log rank test and Cox regression analysis showed that *GBA1*_NMC_ reached cognitive endpoints as defined by the MoCA (GBA1_NMC_: median 5 years, 95% CI: 4.1–5.9; vs GBA1_wildtype_: median 7 years, 95% CI: 6.5–7.5; *p* < 0.001) and the CERAD-Plus (GBA1_NMC_: median 7 years, 95% CI: 5.9–8.1; vs GBA1_wildtype_: median 8 years, 95% CI: 7.1–8.9 ; *p* = 0.001) significantly earlier compared to the *GBA1*_wildtype_ group (Fig. 1 B, C). There was no difference in the clinical endpoint for motor function based on the MDS-UPDRS III total score (Fig. 1 A, *p* = 0.151).

### Incidence of PD and characteristics of PD converters

Five out of the 56 GBA1_NMC_ (8.9%; 3 GBA1_risk__and 2 GBA1_mild_) were diagnosed with PD according to clinical diagnostic criteria defined by classical PD motor symptoms in the course of the study whereas in the GBA1_wildtype_ PD was diagnosed in 2 out of 112 participants (1.8%; *p* = 0.004). One GBA1-PD converter exhibited clinical characteristics of dementia with lewy bodies (DLB) already at baseline, consequently being excluded from the longitudinal analyses.

Descriptive characteristics of PD converters of the GBA1_wildtype_ and GBA1_NMC_ groups at baseline are included in Table 1 showing that GBA1-PD converters were slightly older than GBA1_NMC_ and GBA1_wildype_. There was only one female GBA1-PD converter. Family history of PD, years of education and MDS-UPDRS total score as well as MDS-UPDRS tremor, bradykinesia and PIGD subscores were higher in GBA1_NMC_ than in all other groups. Quantitative motor markers did not reveal any notable differences. MMSE, MoCa and CERAD-Plus sum scores as well as CERAD-Plus subtest showed comparable results compared to the other groups. NfL was remarkably higher than in the GBA1_NMC_ group, but only slightly higher compared to the GBA1_wildtype_ group.

Excluding all PD converters from the LMEM and Kaplan–Meier analyses did not relevantly influence the results.

## Discussion

This study provides comprehensive longitudinal evaluation of GBA1_NMC_ leveraging data of an assessment battery covering a broad panel of non-motor markers, motor and cognitive function as well as serum NfL levels of the largest cohort of GBA1_NMC_ with the longest follow-up of up to 9 years to date.

Our findings indicate that GBA1_NMC_ compared to age- and sex-matched GBA1_wildtype_ show (i) worse performance in global cognitive function as well as in the subdomains of executive and memory function at baseline, (ii) faster longitudinal progression to clinical endpoints of cognitive performance defined by the MoCA and the CERAD-Plus battery, (iii) worse motor performance in Pegboard and 20 m fast walking at baseline, and a higher annual increase of the MDS-UPDRS III bradykinesia subscore, (iv) a higher prevalence of conversion to PD. However, performances in the MDS-UPDRS III total score at baseline as well as longitudinally were comparable, as were ratings of classical non-motor markers (except cognition) and sNfL levels. Surprisingly, a positive family history of dementia was more frequent in the GBA1_wildtype_ group, which might be due to the high motivation of healthy individuals with a positive family history to participate in the TREND study as the study was explicitly designed and promoted to provide early detection of Parkinson’s disease and Alzheimer’s Dementia.

In summary, the faster progression to clinical endpoints of cognitive decline in the GBA1_NMC_ group seems to characterize the profile of GBA1_NMC_.

In line with our findings, the two largest studies investigating GBA1_NMC_ to date leveraging cross-sectional data from the *Parkinson’s Progression Marker Initiative* (PPMI) study^16^ and from a large Gaucher disease center^17^ showed higher MDS-UPDRS III and lower MoCA scores in GBA1_NMC_, but inconsistent differences in other non-motor features (e. g. RBD, mood and olfaction) compared to GBA1_wildtype_. Focusing on a more detailed investigation of cognitive function, a cross-sectional study has recently shown that executive function assessed by the Stroop test was worse in GBA1_NMC_ compared to GBA1_wildtype_ and that reduced global cognitive function based on the MoCA clustered with hyposmia. Contrary, overall motor score, presence of RBD or depression were similar between groups^15^. However, there is still only sparse longitudinal data available on the evolution of non-motor, motor, and fluid biomarkers in GBA1_NMC_. Three studies published by the same group with 2–6 years of follow-up data of a combined cohort of heterozygous GBA1_NMC_ and biallelic Morbus Gaucher patients with subgroup analyses of the subgroup of heterozygous GBA1 cohort alone, found more deterioration in scales of depression, RBD, olfaction, global cognition (MoCA) as well as MDS-UPDRS part II and III scores in the GBA1_NMC_ group compared to GBA1_wildtype_^12–14^.

All these studies consistently highlight cognitive performance of GBA1_NMC_ as a key marker while motor and other non-motor signs have been shown to be affected in some but not in all investigations. This is of high relevance as clinical trials planned for GBA1_NMC_ need to incorporate cognitive testing as a predictor and an outcome measure. While the MoCA as overall cognitive assessment seems sensitive to detect differences on a group level between GBA1_NMC_ and GBA1_wildtype_, the field needs more data on comprehensive longitudinal cognitive test batteries of all relevant cognitive domains (attention, executive, memory, visuospatial) in order to estimate effect sizes of cognitive decline per year. Notably, subgroup analysis stratified by mutation severity as well as phenoconversion to PD and importantly also to DLB should be taken into account. These data will help to define cognitive outcome measures either per domain or as a composite score across domains and estimate sample sizes for a clinical trial.

In contrast to cognition, trajectories of the other assessed motor and non-motor markers as well as sNfL levels, rather developed in parallel and were primarily associated with time of follow-up and age. This seems to indicate that dynamics of these markers might be primarily associated with age. Also, the clinical tests used to assess these markers might not be sensitive enough and/or the analyzed cohort too small detect subtle early changes.

While there is increasing evidence for the utility of sNfL as a biomarker for disease progression in clinically established PD, sNfL seems not to be a sensitive marker in the non-manifest stage of PD. This is supported by evidence from a recent study of our group in a cohort of incident sporadic PD cases from the TREND study showing that sNfL levels are increased only shortly before the time point of conversion to clinically established PD^18^.

With the development of seed amplification assays (SAA) for the detection of disease-specific misfolded α-synuclein aggregates in various biospecimens, new options to identify subjects at risk on an individual levels have arisen enabling to establish biomarker-defined cohorts at-risk for PD^19,20^. It will be important to assess the evolution of motor, non-motor and fluid biomarkers in individuals who show a positive α-synuclein seeding answer in SAA.

Summarizing the results of our study, there is a great need to define and evaluate novel endpoints and outcomes for clinical trials of GBA1_NMC_. Single motor measures – even assessed with quantitative tools - and a variety of non-motor markers (except cognition) do not seem to be sensitive enough to consistently detect subtle changes in GBA1_NMC_. Therefore, in addition to the established endpoint of conversion to motor PD, it seems reasonable to seriously consider cognitive endpoints as additional outcomes for clinical trials and studies of GBA1_NMC_, in particular given that GBA1 mutations not only confer risk for motor PD but also for DLB as well as cognitive decline eventually resulting in dementia.

Notably, the risk of conversion might be different between mutation severity and age with those carrying severe mutations being younger whereas those with risk variants resemble idiopathic PD in term of age at onset.

Finally, our study with the – to date – longest longitudinal follow-up of GBA1_NMC_ of up to 9 years demonstrates that even in genetically-defined at-risk populations larger, multicenter studies with higher numbers of carriers of severe GBA1 mutations and even longer follow-up periods are highly warranted and might be necessary to delineate trajectories of motor, non-motor and fluid biomarkers to predict conversion to PD and/or cognitive decline and to inform clinical trials that target GBA1.

We acknowledge the following limitations: (i) Our study is of exploratory nature and therefore, our findings need validation in prospective studies of even larger cohorts of *GBA1*_*NMC*_. In this context, stratification by mutation severity will be highly interesting. (ii) We had only a small number of PD converters defined by classical motor symptoms, which limits more sophisticated analysis such as principal component analysis of this specific subgroup. However, with ongoing follow-ups of the TREND study the number of PD converters might further increase yielding more valuable longitudinal data to delineate predictors of conversion to motor PD and cognitive decline. (iii) The group of GBA1_NMC_ only included 3 individuals with severe *GBA1* mutations so that a balanced and robust subgroup analysis by mutation severity was not possible. However, we argue that our findings would be even more pronounced with a higher number of individuals with severe GBA1 mutations. (v) As per the inclusion criteria of the TREND study that only recruited individuals older than 50 years of age, potential earlier changes of trajectories might not be detected. And (v) Linear mixed-effects models (LMEM) might be prone to a decrease of statistical power due to drop-out of participants with pronounced worsening of motor and cognitive function in the course of the study. Furthermore, while with LMEM continuous variables are compared over time, the Kaplan–Meier survival analysis is a time-to-event analysis using a defined endpoint as binary variable. This might explain the different results in our longitudinal analyses using these two statistical methods and further highlights the discussion the field has to make in order to design future studies and trials: which are the best outcome analyses to estimate effects but also that represent patient-related outcomes?

We conclude that our study extends data on the non-PD-manifest phase in GBA1_NMC_ indicating early cognitive deterioration as a potentially characteristic feature. Consequently, comprehensive longitudinal assessments of cognitive function including evaluation of cognitive subdomains is crucial to delineate the evolution of early changes in GBA1_NMC_. This might enable a more accurate stratification of GBA1_NMC_ and in turn allow for a more precise definition of trial design and sample size.

## Methods

### Participants

All participants were assessed as part of the TREND study (*Tübingen Risk Evaluation for Neurodegenerative Diseases*)^21^.

The TREND study is a prospective longitudinal study initiated in 2009 with biennial assessments of 1201 elderly participants aged between 50 and 80 years without neurodegenerative diseases. The study is performed at the Department of Neurology and the Department of Psychiatry of the University Hospital Tübingen, Germany comprising a large comprehensive assessment battery with mainly quantitative, unobtrusive measurements. For more details about the TREND study see https://www.trend-studie.de/. Study data are collected and managed using REDCap electronic data capture tools hosted at University of Tübingen^22^.

### Genetic analysis

DNA was isolated from EDTA blood by salting out method and stored at 4 °C. Genetic screening for *GBA1* variants was done by sanger sequencing of all exons of the *GBA1* gene. Naming of *GBA1* variants is based on the new nomenclature for *GBA* variants including the 39-aminoacid residue. In total, we identified 56 participants harboring a variant in the *GBA1* gene (GBA1_NMC_). *GBA1* variant severity was classified in risk variants (GBA1_risk_ *n* = 48: 19 E365K, 26 T408M, 1 T336S, 1 N427K and 1 N427K + T408M), mild (GBA1_mild_ *n* = 5: N409S) and severe mutations (GBA1_severe_ *n* = 3: 1 H294Q, 1 L483P and 1 L483P + E365K) according to established genotype risks reported for PD^23,24^. To overcome age- and sex-related modifying effects within the total TREND cohort, we defined a nested case-control cohort out of the 1201 TREND participants in the relation of 1:2. We included the 56 *GBA1*_*NMC*_ and randomly selected 112 age- and sex-matched healthy individuals without *GBA1* mutation out of the TREND study cohort. All participants underwent genotyping and were also controlled for not carrying pathogenic mutations in the *LRRK2* gene. Furthermore, all PD *converters* were also tested for not carrying pathogenic mutations in the recessive genes *PRKN, PINK* and *DJ1*.

### Clinical investigations and assessments

Each participant underwent a standardized neurological examination by an experienced movement disorder specialist. Individuals with an incident diagnosis of PD at baseline according to the UK Brain Bank Criteria were excluded from the present analysis. Individuals who developed PD during the follow up period were excluded from the longitudinal analyses after the time point of their respective diagnosis.

Family history for PD and dementia, and years of education were assessed with standardized questionnaires. The German version of the Beck’s Depression Inventory II (BDI-II)^25^ was used to assess depressive symptoms. The RBD screening questionnaire (RBDSQ)^26^ was used to assesses sleep behavioral symptoms. Olfactory function was investigated with the 16 Sniffin’ Sticks test^27^. Autonomic symptoms, specifically orthostatic, urinary, and erectile dysfunction as well as constipation, were assessed using subitems 9 to 12 of the Unified Multiple Systems Atrophy Rating Scale (UMSARS)^28^.

Global cognitive function was assessed with the Mini Mental Status Examination (MMSE)^29^ and the Montreal Cognitive Assessment (MoCA)^30^. Since the MoCA was not available until 2009, MMSE scores from all visits of all patients were additionally converted into MoCA equivalent scores using a published algorithm^31^.

Detailed cognitive testing was performed using the extended German version of the *Consortium to Establish a Registry for Alzheimer’s Disease-Plus* (CERAD-Plus)^32^. The neuropsychological CERAD-Plus battery assesses 4 cognitive domains with the following respective subtests (in brackets): executive function (Trail Making Test [TMT] part B, semantic and phonemic verbal fluency), memory (word list learning, word list recall and figure recall), language (Boston Naming Test) and visuospatial function (Figure copy). Additionally, part A of the TMT was performed to assess psychomotor speed. Age, gender, and education adjusted z-scores were used.

Severity of motor symptoms was assessed by the MDS-UPDRS III. Additionally, subscores for tremor, rigidity, bradykinesia and postural instability-gait difficulty (PIGD) were calculated from the respective subitems of the MDS-UPDRS III as described before^33,34^. Purdue Pegboard was used for examination of hand dexterity and combined performance of fine motor speed and finger-eye coordination^35^. Gait speed was assessed quantitatively with the 3-meter Timed Up and Go Task (3 m TUG)^36^ and walking of a straight 20 m track with normal and fast speed as well as fast speed walking while making crosses and serial subtractions of 7 starting from 100 respectively.

Clinical endpoints were defined for motor and cognitive function according to established cut-offs. Motor deterioration reflecting subthreshold parkinsonism was assessed using the MDS-UPDRS III with a cut-off of >6 points excluding scores of the postural and action tremor items^37^. Cognitive endpoints for Mild Cognitive Impairment (MCI) were defined as (i) <26 points in the MoCA score as established^38^ and (ii) a decline of >0.03 based on the mean of the z-normalized CERAD-Plus total score as described recently^39^.

### Serum Neurofilament light chain (sNfL)

Serum NfL levels were measured in duplicates by single-molecule array (SIMOA) technique on the Simoa HD-1 Analyzer (Quanterix, Lexington, Massachusetts), as established previously^40^.

#### Statistical analysis

Statistical analysis was performed using SPSS statistical software version 28.0 (IBM Corp., Armonk, NY) and RStudio software (release 2021.09.02 + 382) using R version 4.1.2 for data visualization. Analyses of cross-sectional data were performed using Student’s *t* test for continuous data and χ^2^ test for categorial variables. All statistical tests were two-sided and *p* values ≤ 0.05 were considered statistically significant. As all analyses were explorative, we did not correct for multiple testing.

Longitudinal analyses using linear mixed-effects models (LMEM) adjusting for age and years of education were performed to estimate the slopes of motor and non-motor parameters and NfL with the fixed factors group (GBA1_wildtype_, GBA1_NMC_) and time (time of follow-up in years from baseline), their interaction and the random variable subject, modeled by random intercepts. We analyzed the fixed effect of group, time and the interaction of group and time on the dependent variable, respectively. Kaplan-Meyer survival curves with log rank test and Cox regression analyses adjusted for age were used to estimate disease-free event of the defined motor and cognitive endpoints.

#### Ethical standards

The study was performed in accordance with the ethical standards laid down in the 1964 Declaration of Helsinki and its later amendments. Ethical approval of the study was granted by the ethical committee of the University of Tübingen (Nr. 90/2009BO2) and written informed consent from all participants was obtained prior to study inclusion.

### Reporting summary

Further information on research design is available in the Nature Research Reporting Summary linked to this article.

### Supplementary information

Reporting Summary

## Data Availability

Anonymized data are available upon request to: benjamin.roeben@med.uni-tuebingen.de
